# Oridonin Dose-Dependently Modulates the Cell Senescence and Apoptosis of Gastric Cancer Cells

**DOI:** 10.1155/2021/5023536

**Published:** 2021-11-09

**Authors:** Yiping Wang, Hang Lv, Chunyan Dai, Xi Wang, Yifei Yin, Zhe Chen

**Affiliations:** Key Laboratory of Digestive Pathophysiology of Zhejiang Province, the First Affiliated Hospital of Zhejiang Chinese Medical University, 54 Youdian Road, Hangzhou 310006, China

## Abstract

Gastric cancer (GC) is the fourth most lethal cancer. Effective treatments are lacking, and our knowledge of the pathogenic mechanisms in play is poor. Oridonin from the Chinese herb *Rabdosia rubescens* exerts various anticancer activities. However, the dose-dependent effects of oridonin on human GC remain unclear. Here, we found that oridonin inhibited GC cell growth in a time- and dose-dependent manner. Low-dose oridonin induced GC cell cycle arrest at G0/G1 and cell senescence by suppressing the c-Myc-AP4 pathway and enhancing p53-p21 signaling. AP4 overexpression partly abrogated the oridonin-induced senescence of GC cells. High-dose oridonin induced apoptosis and autophagy, with the autophagy inhibitor BafA1 attenuating oridonin-induced apoptosis. Together, the findings indicate that oridonin at different doses modulates GC cell senescence and apoptosis; oridonin may thus usefully treat GC.

## 1. Introduction

Gastric cancer (GC) is the fifth most common cancer worldwide (5.6% of all new cancers) and the fourth leading cause of cancer-related mortality (7.7% of cancer deaths in 2020) [1]. GC incidence varies worldwide, being the highest in Asia, Eastern Europe, and South America [2]. Over the past 50 years, GC incidence and mortality have declined as medical and health standards improved [3]. However, most GCs are diagnosed at advanced stages, and only approximately 25% of patients can tolerate surgical cancer removal. The principal treatment is gastrectomy combined with chemotherapy and radiation therapy [4–7]. Our poor understanding of GC pathogenesis and the lack of effective treatments render early difficulty in diagnosis and high mortality. The mechanisms in play and novel drugs are receiving increasing attention.

**Figure 1:**
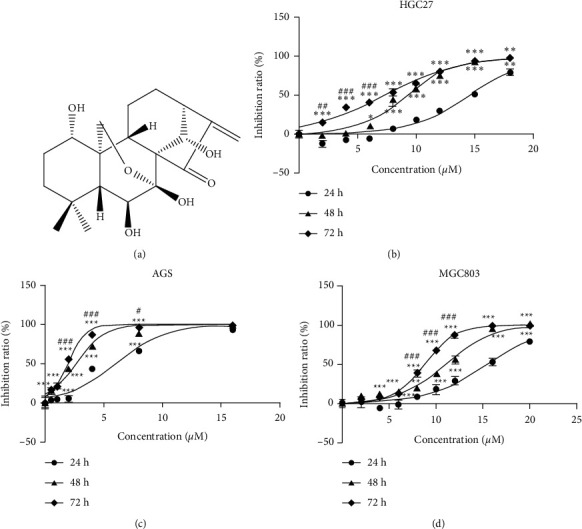
Oridonin inhibited gastric cancer (GC) cell proliferation in vitro. (a) Chemical structure of oridonin. GC cell line HGC27 (b), AGS (c), and MGC803 (d) cells treated with oridonin (0–40 *μ*M) for 24, 48, and 72 h, and cell proliferation was measured using the CCK-8 assay. The data are shown as the mean ± SD of triplicate samples. Analyses were performed using two-way ANOVA. ^*∗∗*^*P* < 0.01; ^*∗∗∗*^*P* < 0.001 (vs. 24 h); ^##^*P* < 0.01; ^###^*P* < 0.001 (vs. 48 h).

Traditional Chinese medicine (TCM) has been used for thousands of years. Many medicinal plants can be taken as food. The homology of Chinese medicine and food ensures the safety and available source. Recent years, with the development of analytical techniques, increasing bioactive compounds from natural sources have been identified, which makes more opportunities in discovery of anticancer drugs. It was reported that more than half of the small-molecule drugs utilized in the area of cancer were from natural sources rather than totally synthetic over the time frame from 1946 to 2019 [8, 9]. Owing to the concerted efforts by scientists, many medicinal plant extracts, such as paclitaxel and hydroxycamptothecin, have been used in clinical treatment of cancer [10]. Oridonin, from the Chinese herb *Rabdosia rubescens*, is a kaurene diterpenoid (Figure 1(a)) that exhibits activity against pancreatic, breast, prostate, and lung cancers and leukemia [11–16]. Oridonin activates apoptosis, cell senescence, and autophagy by modulating the PI3K/AKT, JNK, Wnt/*β*-catenin, TGF-*β*1/Smads-PAI-1, mTOR, and Notch signaling pathways [15–20]. However, the low oral bioavailability (less than 5%) and imprecise mechanisms of oridonin have greatly impacted its potential clinical implications [21]. Interestingly, it was reported that oridonin induces L929 cell apoptosis by regulation of reactive oxygen species-mediated signaling pathways and also simultaneously block apoptosis by inducing autophagy via regulation of p38/NF-*κ*B signaling [22]. These paradoxical results may be associated with cell types and dose-dependent regulation of various signaling pathways in cells. However, the effects of oridonin on GC cells remain poorly known, as does any dose response.

Apoptosis and cellular senescence are two different anticancer responses. After irreversible cell cycle arrest, cells enter senescence, becoming larger and flatter, and produce senescence-associated *β*-galactosidase (SA-*β*-gal) [23]. Apoptosis (regulated cell death) features cell shrinkage and blebbing, as well as chromatin condensation [24]. Oridonin induces apoptosis and senescence of colorectal cancer cells [25, 26], but the mechanisms in play remain unclear, as does whether oridonin acts on GC cells. Here, we used AGS, HGC27, and MGC803 GC cell lines to explore the anticancer effects of oridonin. At low concentrations, oridonin induced cell cycle arrest and cellular senescence by suppressing the c-Myc-AP4 axis. At high concentrations, oridonin induced apoptosis via autophagy. AP4 overexpression partly abrogated oridonin-mediated senescence.

## 2. Materials and Methods

### 2.1. Cell Lines

Human GC cell lines HGC27, MGC803, and AGS were purchased from the Cell Bank of the Chinese Academy of Science (Shanghai, China). Cells were cultured in RPMI-1640 (Gibco, Nuoyang Biotech Co., Ltd., Hangzhou, China) supplemented with 10% fetal bovine serum (Hyclone) at 37°C and 5% CO_2_.

### 2.2. Reagents

Oridonin (empirical formula, C_20_H_28_O_6_; molecular weight, 364.43) was purchased from ChemFaces (Wuhan, China). The purity of the oridonin was confirmed by HPLC to be > = 98%. The oridonin was initially dissolved in dimethyl sulfoxide (DMSO) to make a 20 mM stock solution and stored at −20°C for further use. 0.25% trypsin containing EDTA and fetal bovine serum (FBS) was obtained from Gibco (USA). Cell Counting Kit-8 (CCK-8) was purchased from Dojindo (Japan). PE Annexin V apoptosis detection kit was from BD Biosciences (San Diego, CA, USA). Cell cycle analysis kit including RNase A and propidium iodide (PI) was purchased from Liankebio (Zhejiang, China). The antibodies against p21, cleaved caspase-3, cleaved PARP, GAPDH, *β*-actin, LC3B, anti-rabbit/mouse IgG, and HRP-linked antibody were purchased from CST (Danvers, MA, USA). c-Myc and p53 were purchased from Abcam (Cambridge, UK), and AP4 (6B1) was from Abnova (Taipei, Taiwan).

### 2.3. Cytotoxicity Assay

Cell Counting Kit-8 (CCK-8) was applied to measure the in vitro cytotoxicity of oridonin to GC cells. Briefly, 5 × 10^3^ cells per well were plated in 96-well plates the day before. The medium containing oridonin or DMSO (diluent) at different concentrations was replaced and maintained for 24, 48, or 72 h. Then, 100 *μ*L of the fresh medium in addition to 10 *μ*L CCK-8 solution was used to replace the medium in each well. Absorbance was determined at 450 nm using a spectrophotometer (BioRad, USA).

### 2.4. Colony Formation Assay

Cells were seeded in a 24-well plate and cultured with oridonin. After 10 days, the cells were fixed with methanol for 5 min, dried, and stained with 0.1% crystal violet. The percentage of area covered by cells per view was calculated with Image *J*.

### 2.5. Cell Cycle Analysis

The cells treated with oridonin or DMSO at the indicated concentration for 24 h were collected and fixed with 75% cold ethanol. 200 *μ*L RNase A (1 mg/mL) together with 500 *μ*L of PI (100 *μ*g/mL) were used to stain the cells for 30 min at room temperature; then, the cells were analyzed using a FACStar flow cytometer (BD, Canto II).

### 2.6. Annexin V/PI Staining

Apoptosis was measured by using the PE Annexin V apoptosis detection kit (BD Biosciences, San Diego, CA). Briefly, cells were treated with or without oridonin for 24 h, collected, and washed with cold PBS twice. The cells were analyzed with a FACStar flow cytometer (Canto II) after being resuspended and stained with 400 *μ*L of Annexin V binding buffer, 5 *μ*L of PE Annexin V, and 5 *μ*L of 7-AAD viability staining solution.

### 2.7. Cell Senescence Assay

Senescence-associated expression of *β*-galactosidase activity was assayed with a senescence detection kit (Cell Signaling Technology) on cells treated with DMSO or oridonin at the indicated concentration. The development of cytoplasmic blue was detected and visualized using a light microscope at low magnification.

### 2.8. SDS Polyacrylamide Gel Electrophoresis (SDS-PAGE) and Western Blot Analysis

The cells were lysed with RIPA buffer (CWBio, China) supplemented with protease inhibitor cocktail and phenylmethanesulfonyl fluoride (PMSF). Equal amounts of proteins were separated by SDS-PAGE and transferred to polyvinylidene fluoride membranes (PVDF, Life Sciences). Immunoblots were visualized by the enhanced chemiluminescence Western blot substrate kit (4A Biotech, China), and images were obtained by the ChemiDoc Touch Imaging System (BioRad, USA).

### 2.9. Immunofluorescence Staining

Cells were fixed in 4% paraformaldehyde for 15 min, permeabilized with 0.2% Triton X-100 for 15 min, and then blockade with 5% goat serum for 1 h at room temperature. Then, cells were incubated with primary antibody (anti-LC3B) overnight and the corresponding secondary antibody, and the nuclei were stained with DAPI. Stained cells were imaged using a Leica SP8 confocal fluorescence microscope.

### 2.10. Gene Overexpression

The AP4 expression vector was constructed by inserting its ORF sequence into the GV341 vector (GeneChem). The vector lacking an insert for AP4 overexpression was used as control. All insertions were further confirmed by DNA sequencing. Lentivirus preparations were produced and supplied by the Shanghai GeneChem, Co., Ltd., China. AGS and MGC803 cells were infected with AP4 lentivirus in addition of lentivirus into the cell culture at MOI of 100 and 50, respectively. After 3 days of infection, cells were screened with puromycin for one week and then ready for use.

### 2.11. Statistical Analysis

The data were presented as mean ± standard deviation (SD). Statistical comparisons were performed using GraphPad Prism version 5.0 (GraphPad Software, San Diego) for variance (ANOVA). Student's *t*-test was used to analyze differences between two samples means. *P* < 0.05 was considered statistically significant.

## 3. Results

### 3.1. Oridonin Suppressed GC Cell Proliferation and Induced GC Cell Cycle Arrest

**Figure 2:**
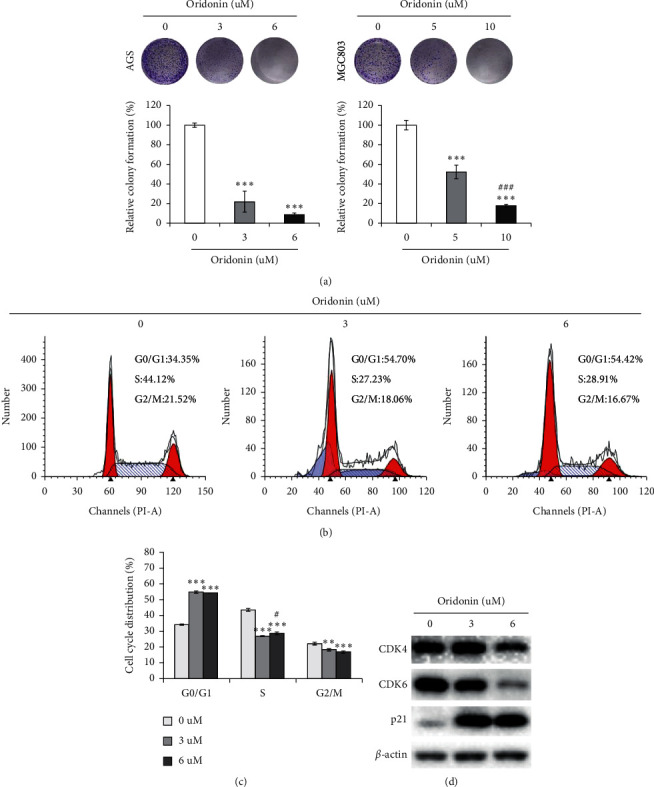
Oridonin induced cell cycle arrest in GC cells. (a) Colonies of MGC803 and AGS cells after treated with low-dose oridonin. Data are expressed as mean ± SD (*n* = 3; ^*∗∗*^*P* < 0.01; ^*∗∗∗*^*P* < 0.001 (vs. 0 *μ*M); ^###^*P* < 0.001 (vs. 5 *μ*M)). (b) AGS cells maintained in medium containing gradient concentrations of oridonin for 24 h. The cell cycle distribution is analyzed by flow cytometric assay. (c) Quantitative analysis of cell cycle distribution. Data are presented as the mean ± SD of three separate experiments. ^*∗∗*^*P* < 0.01; ^*∗∗∗*^*P* < 0.001 (vs. 0 *μ*M); ^#^*P* < 0.05 (vs. 3 *μ*M) (d) AGS cells treated with oridonin for 24 h cells and subjected to Western blot analysis. The expression levels of CDK4, CDK6, and p21 were analyzed; *β*-actin is used as a protein-loading control.

AGS, HGC27, and MGC803 GC cells were treated with oridonin (0–40 *μ*M) for 24, 48, and 72 h, and cell proliferation was measured using the CCK-8 assay. As shown in Figures 1(b)–1(d), oridonin suppressed GC cell proliferation in a time- and dose-dependent manner. The IC_50_ values were given in Table 1. After treatment with oridonin for 10 days, colony formation by AGS and HGC27 cells was significantly inhibited (Figure 2(a)). Flow cytometry (Figures 2(b) and 2(c)) showed that oridonin increased the proportion of AGS cells in the G0/G1 phase and decreased those in the S and G2/M phases. The expression levels of cell cycle-related proteins (p21, CDK4, and CDK6) were measured using Western blotting. Oridonin increased p21 expression but reduced CDK4 and CDK6 expression (Figure 2(d)).

### 3.2. Low-Dose Oridonin Induced GC Cellular Senescence

**Figure 3:**
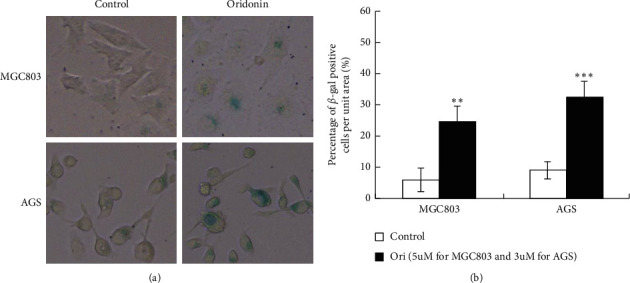
Low-dose oridonin induced GC cellular senescence. (a) MGC803 and AGS cells treated with 5 and 3 *μ*M oridonin, respectively, for 48 h and then maintained cells with oridonin-free medium for additional 4 days. Senescent cells are identified by senescence-associated *β*-galactosidase activity assay. (b) Quantitative analysis of senescent cells with the percentage of *β*-gal-positive cells per unit area (*n* = 3, bars are the mean ± SD, ^*∗∗*^*p* < 0.01, ^*∗∗∗*^*p* < 0.001).

**Figure 4:**
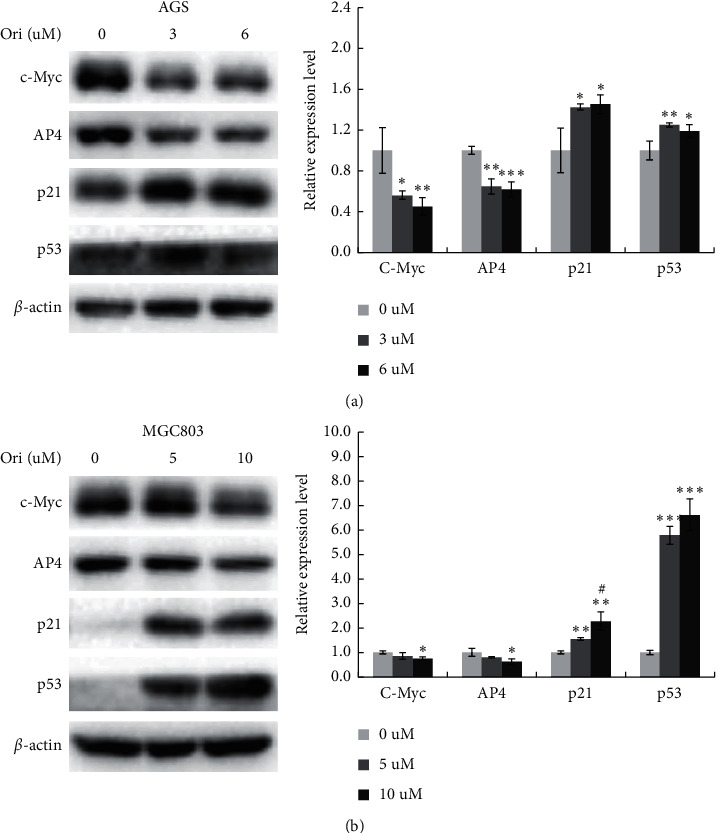
Oridonin regulated the expression of c-Myc, AP4, p53, and p21 in vitro. (a) AGS and (b) MGC803 cells treated with low-dose oridonin for 48 h. Western blot is used to examine the expression levels of c-Myc, AP4, p53, and p21. *β*-Actin is used as a protein-loading control. The graph shows densitometric analysis of the c-Myc, AP4, p53, or p21 relative to *β*-actin (data are presented as the mean ± SD, ^*∗*^*p* < 0.05, ^*∗∗*^*p* < 0.01, ^*∗∗∗*^*p* < 0.001 (vs. 0 *μ*M); ^#^*P* < 0.05 (vs. 5 *μ*M)).

Cellular senescence is irreversible cell cycle arrest that is triggered in response to stress and regarded as an anticancer consequence of chemotherapy [27].We exposed AGS and MGC803 cells to 3 and 5 mΜ oridonin, respectively, for 48 h, followed by 4 days of oridonin-free culture. Oridonin-treated cells expressed SA-*β*-gal, with the percentage of *β*-gal-positive cells increasing from 9.1% to 32.5% and 6.0% to 24.6% in AGS and MGC803 cells, respectively (Figures 3(a) and 3(b)). The p53-p21^Cip1^ pathway is a key mediator of signaling involved in cellular senescence [28]; we thus measured p53 and p21 expression levels and those of c-Myc and AP4 (upstream regulators of p21) [29]. Western blotting showed that the p53 and p21 levels increased, but those of c-Myc and AP4 decreased, after oridonin treatment (Figures 4(a) and 4(b)), suggesting that p53-p21^Cip1^ signaling played an important role in the oridonin-induced cellular senescence.

**Figure 5:**
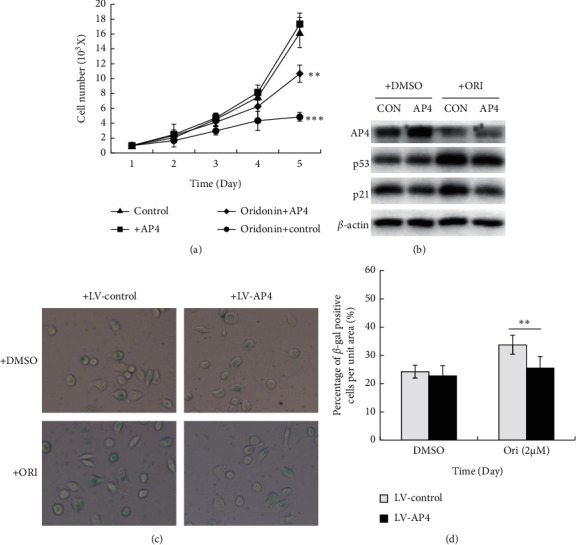
AP4 overexpression reversed the oridonin-induced senescence of GC cells. (a) AGS cells are infected with AP4 virus or control virus by addition of virus into the cell culture at MOI of 100. The proliferation of AGS cells that overexpressed AP4 or the negative control treated with or without oridonin is determined. ^*∗∗*^*P* < 0.01 and ^*∗∗∗*^*P* < 0.001. The *P* values are analyzed by two-way ANOVA. (b) AGS cells that infected with AP4virus or control virus are treated with or without oridonin, Western blot analysis is performed to examine the expression of AP4, p53, and p21 in cells. *β*-Actin is used as a protein-loading control. (c) AGS cells infected with AP4 virus or control virus are treated with or without oridonin for 48 h and then maintained cells with oridonin-free medium for additional 4 days. *β*-gal-positive cells are identified by senescence-associated *β*-galactosidase activity assay. (d) Quantitative analysis of senescent cells with the percentage of *β*-gal-positive cells per unit area (*n* = 3, bar graphs are plotted as the mean ± SD, ^*∗∗*^*p* < 0.01).

We explored whether oridonin-mediated AP4 downregulation explained the senescence of GC cells. The Western blots in Figures 5(a) and 5(b) show that, compared to cells treated with a control virus, the AP4 expression level increased by 169.5% on transformation of an AP4-encoding lentivirus into AGS cells, but cell proliferation was not affected. AP4 overexpression partly rescued oridonin-suppressed cell proliferation. After oridonin treatment, the proportion of *β*-gal-positive AP4-expressing cells was much lower than that of control cells, indicating that the former cells exhibited an enhanced proliferation capacity (Figures 5(c) and 5(d)). After treatment with oridonin, the p53 and p21 levels were higher in AGS cells infected with the control virus than the AP4-encoding lentivirus (Figure 5(b)). We also used the other cell line (MGC803 cells) to verify the effects of overexpressing AP4 on oridonin-induced senescence, and the similar trends appeared in MGC803 cells. As shown in Supplementary Figure 1, compared to cells treated with a control virus, overexpression of AP4 alleviated the inhibition of cell proliferation, lowered the p53 and p21 levels, and significantly lightened the proportion of *β*-gal-positive cells after oridonin treatment. Thus, AP4 downregulation contributed (at least in part) to the oridonin-induced inhibition of the proliferation and senescence of GC cells.

### 3.3. High-Dose Oridonin Induced GC Cell Apoptosis

**Figure 6:**
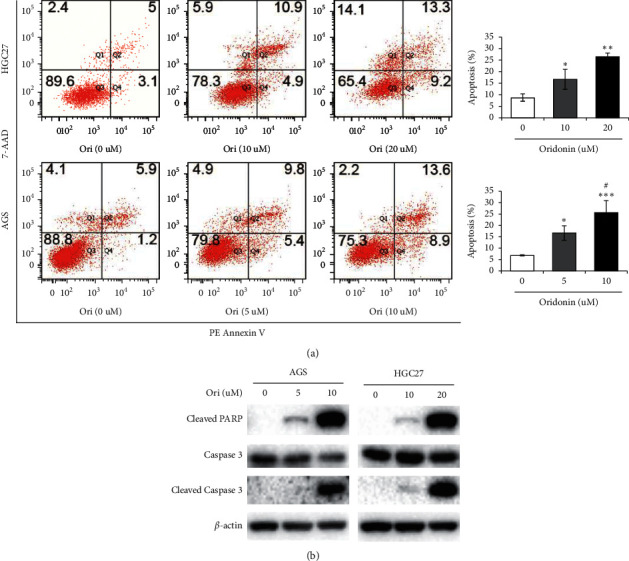
High-dose oridonin induced GC cell apoptosis. (a) HGC27 and AGS cells treated with oridonin for 24 h. The cells were stained with 7-AAD and PE Annexin V. Apoptotic cells were identified using flow cytometry. Data are presented as the mean ± SD of three independent experiments (^*∗*^*p* < 0.05, ^*∗∗*^*p* < 0.01, ^*∗∗∗*^*p* < 0.001 (vs. 0 *μ*M); ^#^*p* < 0.05 (vs. 5 *μ*M)). (b) Effects of oridonin on the expression of apoptotic-associated proteins. MGC803 and AGS GC cells were treated with oridonin for 24 h. Western blot analysis was carried out with antibodies against cleaved PARP, caspase-3, and cleaved caspase-3. *β*-Actin was used as a protein-loading control.

Given that apoptosis inhibits cancer progression, we tested the effects of high-dose oridonin on GC cells. AGS and HGC27 cells were exposed to oridonin for 24 h, and then, the cells were collected and stained with 7-AAD and PE Annexin V. Apoptotic cells were identified using flow cytometry. As shown in Figure 6(a), oridonin increased the apoptosis rate of both HGC27 and AGS cells. The proportions of apoptotic HGC27 cells were as follows: control, 8.77 ± 1.51%; 10 *μ*M oridonin, 16.63 ± 4.31% (*P* < 0.05); and 20 *μ*M oridonin, 26.33 ± 1.77% (*P* < 0.001). For AGS cells, the figures were control, 6.80 ± 0.30%; 5 *μ*M oridonin, 16.60 ± 3.23% (*P* < 0.01); and 10 *μ*M oridonin, 25.53 ± 3.54% (*P* < 0.01). Western blotting (Figure 6(b)) showed that oridonin (especially at high doses) significantly upregulated the levels of cleaved caspase-3 and cleaved PARP.

### 3.4. Oridonin Triggered Apoptosis by Inducing Autophagy

**Figure 7:**
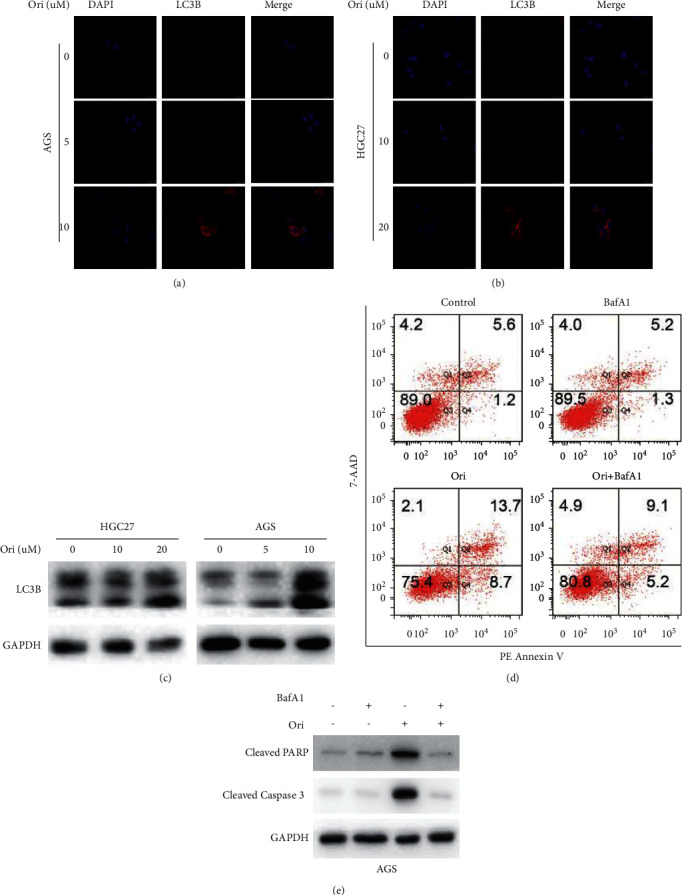
Oridonin triggered apoptosis by inducing autophagy in GC cells. The expression of LC3 in the AGS (a) and HGC27 (b) cells exposed to oridonin was compared by immunofluorescence and Western blot; (c) the expression of LC3 examined by Western blot. (d)-(e) AGS cells are treated with high-dose oridonin, with or without pretreated with BafA1 (0.5 nM, 12 h), and then, the apoptosis and expression of apoptotic markers in AGS cells are assayed.

Both apoptosis and autophagy kill cancer cells, with autophagy sometimes triggering apoptosis [30, 31]. Immunofluorescence assays revealed that cellular LC3 puncta accumulated after AGS and HGC27 cells were exposed to high (but not low) levels of oridonin, as shown in Figures 7(a) and 7(b). Western blotting showed that oridonin increased LC3B expression in GC cells in a dose-dependent manner (Figure 7(c)). We used bafilomycin A1 (BafA1) (an inhibitor of autophagy) to explore whether oridonin induced apoptosis via autophagy. As shown in Figures 7(d) and 7(e), oridonin-induced apoptosis was reversed by autophagy blockade with BafA1, and BafA1 pretreatment decreased the oridonin-induced increases in the cleaved PARP and cleaved caspase-3 levels. Thus, oridonin induced GC cell apoptosis by activating autophagy.

## 4. Discussion

Oridonin is first isolated from *Rabdosia rubescens*, which also has a traditional Chinese medicine name “Donglingcao.” Donglingcao is a commonly available over-the-counter (OTC) herbal medicine for the treatment of inflammation and cancer in China [32–34]. Oridonin, as one of the main active components of Donglingcao, exhibits a promising safety profile and potent antitumor activities [11–20]. In view of its good stability, large commercially available supply, and suitable molecular weight, oridonin is a potential anticancer drug candidate. However, the disadvantages of oridonin, including limited bioavailability and the vague mechanisms of action, hinder its further clinical application. In light of the different phenotype and regulatory mechanism of oridonin in types of tumor cells, we aimed to figure out the effects and dose response of oridonin on GC cells. The results showed that oridonin inhibited the growth of HGC27, MGC803, and AGS GC cells in a time- and dose-dependent manner (Figure 1).

Apoptosis is programmed cell death, and senescence is permanent cell cycle arrest. Senescence is triggered at much lower drug doses than those required to induce apoptosis, significantly reducing the side effects of anticancer therapy and thus improving quality of life [35]. For example, low-dose resveratrol inhibited the growth of lung cancer cells and induced premature senescence by increasing reactive oxygen species-mediated DNA damage [36]. We found that oridonin inhibited GC cell colony formation in a dose-dependent manner and induced cell cycle arrest at G0/G1 (Figure 2). Low-dose oridonin significantly increased SA-*β*-gal staining and elevated the p53 and p21 levels of GC cells (Figures 3-4). c-Myc-AP4 downregulation was involved in the inhibition of GC cell proliferation and senescence induced by oridonin. c-Myc plays important roles in human cell growth, metabolism, and tumorigenesis [37]. Myc inactivation induced tumor regression by triggering the senescence of lymphoma and hepatocellular carcinoma cells [38]. AP4 is a ubiquitous transcription factor that plays a major role in mammalian cells [29, 39–41]. AP4 directly represses p16 and p21 expression (both proteins are regulators of cellular senescence), thus suppressing the senescence of AP4-deficient mouse embryo fibroblasts [42]. Moreover, the AP4 gene is a direct transcriptional target of c-Myc, overexpression of which increased the AP4 level in MCF-7 cells [41]. We found that AP4 overexpression attenuated the oridonin-induced senescence of GC cells (Figure 5 and Supplementary Figure 1). However, the proportions of *β*-gal-positive AP4-expressing and control cells were similar, unlike what was noted when AP4 induced the senescence of confluent human retinal pigment epithelium cells via p53 activation [43]. This inconsistency may reflect differences in the culture conditions. Long-term (>20 days) AP4 overexpression in confluent retinal pigment epithelium cells greatly stressed the cells, and oridonin-mediated GC cell senescence was rapid. Therefore, GC cells expressing AP4 did not enter senescence. Also, different cell lines were employed; the global gene expression profiles and signal networks differ between cancer and noncancer cells. Nevertheless, the mechanism of oridonin-induced inhibition of AP4 is worth to be further investigated.

Autophagy, also termed self-digestion, is in play when cells are under stress, starvation, and inflammation. Many of the stimulators can trigger autophagy, apoptosis, or both. Autophagy usually develops prior to apoptosis and may in fact control apoptosis, which has a therapeutic advantage in the prevention and therapy of cancer [30, 31]. Increasing natural bioactive compounds, including resveratrol, baicalin, and thymoquinone, have exhibited antitumor potential through inducing apoptosis or autophagy or both [10, 44, 45]. In this study, we found that oridonin at high concentrations induced GC cell apoptosis and significantly upregulated the levels of cleaved caspase-3 and cleaved PARP (Figure 6). High level of oridonin increased the levels of cellular LC3 puncta in GC cells and that of LC3B (a marker of autophagy); thus, oridonin could activate autophagy in GC cells. After blockade of autophagy with BafA1, the levels of oridonin-induced apoptosis fell, suggesting that high-dose oridonin induced apoptosis via activation of autophagy (Figure 7). The interplay between apoptosis and autophagy is complex, and studies on this topic are often contradictory. The molecular mechanism by which oridonin induced autophagy remains obscure; thus, further work concerning the action mechanism of oridonin on crosstalk between apoptosis and autophagy is needed. In addition, in view of the dose-mediated effects of oridonin in vitro, whether the dose responses of oridonin do in vivo or not warrants further investigation.

## 5. Conclusion

We thus found that oridonin affected GC cells differently at different doses. Low-dose oridonin induced senescence by inactivating the c-Myc-AP4 pathway, whereas high-dose oridonin induced apoptosis by activating autophagy. Oridonin may usefully treat GC.

## Figures and Tables

**Table 1:** IC_50_ of oridonin in gastric cancer cell lines.

Cell line	IC50, *μ*Μ (24 h)	IC50, *μ*Μ (48 h)	IC50, *μ*Μ (72 h)
AGS	5.995 ± 0.741	2.627 ± 0.324	1.931 ± 0.156
HGC27	14.61 ± 0.600	9.266 ± 0.409	7.412 ± 0.512
MGC803	15.45 ± 0.59	11.06 ± 0.400	8.809 ± 0.158

Data are shown as the mean ± standard deviation of triplicate determination. IC_50_, half maximal inhibitory concentration.

## Data Availability

Data are available from the corresponding author upon request.

## Supplementary Materials

Supplementary Materials Supplementary Figure 1. AP4 overexpression reversed the oridonin-induced inhibition of the proliferation and senescence of GC cells. *s*
