# Supplementary material for: Oridonin Dose-Dependently Modulates the Cell Senescence and Apoptosis of Gastric Cancer Cells
Source: Evid Based Complement Alternat Med. 2021 Nov 9;2021:5023536. doi: 10.1155/2021/5023536 (PMC8595004; doi:10.1155/2021/5023536)

Supplementary Information to the manuscript

**Oridonin dose-dependently modulates the cell senescence and apoptosis of gastric cancer cells**

Yiping Wang^1^, Hang Lv^1^, Chunyan Dai^1^, Xi Wang^1^, Yifei Yin^1^, Zhe Chen^1^*

^1^ Key Laboratory of Digestive Pathophysiology of Zhejiang Province, the First Affiliated Hospital of Zhejiang Chinese Medical University, 54 Youdian Road, Hangzhou, 310006, China

To whom correspondence should be addressed: Zhe Chen(chenzhe@zju.edu.cn), Key Laboratory of Digestive Pathophysiology of Zhejiang Province, the First Affiliated Hospital of Zhejiang Chinese Medical University,54 Youdian Road, Hangzhou, 310006, China. Tel.: Tel: +86-571-8660280, Fax: +86-571-86620280.

This file contains one supplementary figure legend and supplementary figure.

**Supplementary figure 1.** **AP4 overexpression reversed the oridonin-induced inhibition of the proliferation and senescence of GC cells.**

(A) MGC803 cells were infected with AP4 virus or control virus by addition of virus into the cell culture at MOI of 50. The proliferation of MGC803 cells that overexpressed AP4 or the negative control were treated with or without oridonin was determined. *** for P<0.001. The *P* values were analyzed by two-way ANOVA. (B) MGC803 cells that infected with AP4virus or control virus were treated with or without oridonin, Western blot analysis was performed to examine the expression of AP4, p53 and p21 in cells, β‑actin was used as a protein‑loading control. (C) MGC803 cells infected with AP4 virus or control virus were treated with or without oridonin for 48 h; then maintained cells with oridonin-free medium for additional 4 days. β-gal positive cells were identified by senescence-associated β-galactosidase activity assay. (D) Quantitative analysis of senescent cells with the percentage of β-gal positive cells per unit area (n = 3, bar graphs are plotted as the mean ± SD, **p* < 0.05).

**Supplementary figure 1**


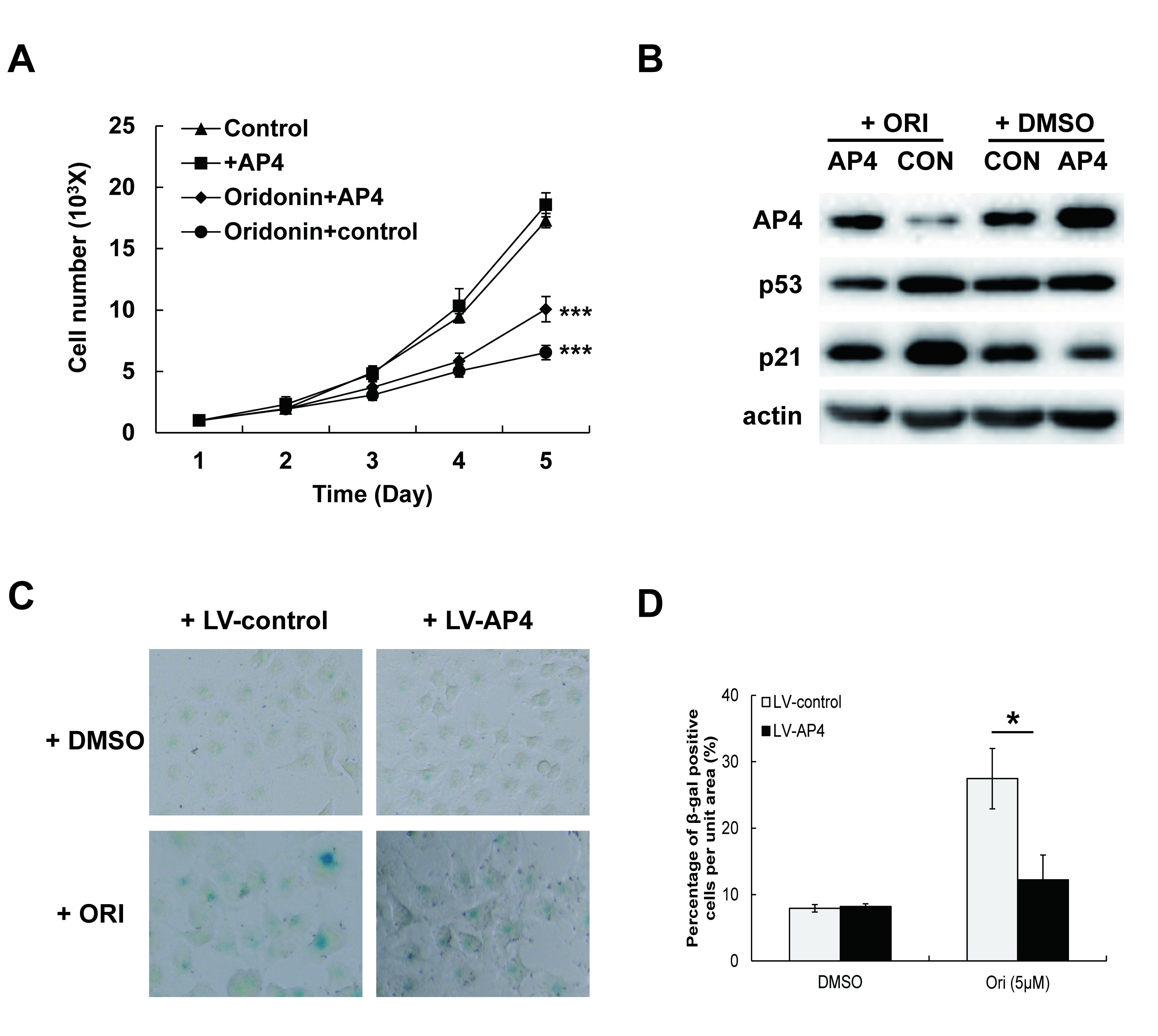

Supplement: Supplementary Materials — Supplementary Figure 1. AP4 overexpression reversed the oridonin-induced inhibition of the proliferation and senescence of GC cells. s [file 5023536.f1.docx]
